# Neurocognitive Changes in Patients with Post-COVID Depression

**DOI:** 10.3390/jcm13051442

**Published:** 2024-03-01

**Authors:** Marina Khodanovich, Anna Naumova, Daria Kamaeva, Victoria Obukhovskaya, Svetlana Vasilieva, Evgeny Schastnyy, Nadezhda Kataeva, Anastasia Levina, Marina Kudabaeva, Valentina Pashkevich, Marina Moshkina, Yana Tumentceva, Mikhail Svetlik

**Affiliations:** 1 Laboratory of Neurobiology, Research Institute of Biology and Biophysics, Tomsk State University, 36 Lenina Ave., Tomsk 634050, Russia; susl2008@yandex.ru (D.K.); diada1991@gmail.com (V.O.); nadi-51@yandex.ru (N.K.); nastasio@yandex.ru (A.L.); kmsra08@gmail.com (M.K.); pashkevich.pvuu97@mail.ru (V.P.); moshkinamv989@gmail.com (M.M.); mimizyana@gmail.com (Y.T.); mihasv@gmail.com (M.S.); 2 Department of Radiology, School of Medicine, South Lake Union Campus, University of Washington, 850 Republican Street, Seattle, WA 98109, USA; nav@uw.edu; 3 Laboratory of Molecular Genetics and Biochemistry, Mental Health Research Institute, Tomsk National Research Medical Center of the Russian Academy of Sciences, 4 Aleutskaya Street, Tomsk 634014, Russia; 4 Department of Fundamental Psychology and Behavioral Medicine, Siberian State Medical University, 2 Moskovskiy Trakt, Tomsk 6340505, Russia; 5 Department of Affective States, Mental Health Research Institute, Tomsk National Research Medical Center of the Russian Academy of Sciences, 4 Aleutskaya Street, Tomsk 634014, Russia; vasilievasn@yandex.ru (S.V.); schastnyy@gmail.com (E.S.); 6 Department of Neurology and Neurosurgery, Siberian State Medical University, 2 Moskovskiy Trakt, Tomsk 6340505, Russia; 7 Medica Diagnostic and Treatment Center, 86 Sovetskaya Street, Tomsk 634510, Russia

**Keywords:** COVID-19, post-COVD-19 condition, long COVID, depression, major depressive disorder, cognitive impairment, MoCA, Word Memory Test, Stroop Color Word Test, Trail Making Test

## Abstract

**Background**: Depression and cognitive impairment are recognized complications of COVID-19. This study aimed to assess cognitive performance in clinically diagnosed post-COVID depression (PCD, n = 25) patients using neuropsychological testing. **Methods**: The study involved 71 post-COVID patients with matched control groups: recovered COVID-19 individuals without complications (n = 18) and individuals without prior COVID-19 history (n = 19). A post-COVID depression group (PCD, n = 25) was identified based on psychiatric diagnosis, and a comparison group (noPCD, n = 46) included participants with neurological COVID-19 complications, excluding clinical depression. **Results**: The PCD patients showed gender-dependent significant cognitive impairment in the MoCA, Word Memory Test (WMT), Stroop task (SCWT), and Trail Making Test (TMT) compared to the controls and noPCD patients. Men with PCD showed worse performances on the SCWT, in MoCA attention score, and on the WMT (immediate and delayed word recall), while women with PCD showed a decline in MoCA total score, an increased processing time with less errors on the TMT, and worse immediate recall. No differences between groups in Sniffin’s stick test were found. **Conclusions**: COVID-related direct (post-COVID symptoms) and depression-mediated (depression itself, male sex, and severity of COVID-19) predictors of decline in memory and information processing speed were identified. Our findings may help to personalize the treatment of depression, taking a patient’s gender and severity of previous COVID-19 disease into account.

## 1. Introduction

Post-COVID syndrome is defined as a state following COVID-19 in people with a probable or confirmed history of infection, usually occurring 3 months after the onset of COVID-19 symptoms and lasting at least 2 months, which cannot be explained by an alternative diagnosis [1]. In September 2020, the World Health Organization introduced the corresponding codes denoting the post-COVID-19 condition, including post-COVID-19 syndrome, International Classification of Diseases (ICD)-10 code (U09), and ICD-11 code (RA02).

To date, a large amount of evidence regarding cognitive and depressive impairments in post-COVID patients has been accumulated [2,3,4,5,6,7,8,9,10,11,12]. SARS-CoV-2 infection is associated with an increased risk of developing mental disorders, including depression, which are detected both during the acute phase and in the post-COVID period. A study by Ma et al. [13] reported that 43.1% of patients showed signs of depression based on data from the online self-questionnaire nine-item Patient Health Questionnaire (PHQ-9) during the acute period of coronavirus infection. A retrospective cohort study by Taquet M. et al., 2021, including 236,379 patients, demonstrated that mood disorders, anxiety, and psychotic disorders as consequences of COVID-19 were detected in 23.98% of people who had the infection [3]. 

A significant proportion of post-COVID patients report depressive symptoms as well as cognitive impairment [2,3,4,5,6,7,8,9,10,12,13,14]. In the published studies on post-COVID patients, data on cognitive impairment and depressive symptoms were mainly obtained through self-assessment questionnaires [6,7,10,11,12,15,16,17]. The main limitations of most of these studies are the lack of a psychiatrist-confirmed diagnosis of depression and the use of objective psychometric tests to assess cognitive function. Self-reported symptoms of depression were shown as the most significant predictor of cognitive impairment [7]. However, depressive symptoms were not confirmed by a psychiatrist. Despite the large number of published reports, we did not find in the available literature studies on cognitive functions in post-COVID patients who were diagnosed with depression by a psychiatrist. Only a few studies used standardized tests for this assessment [4,8,9]. Self-reported symptoms and performances on cognitive tests may differ significantly [18]. To reduce subjectivity and increase precision in the characterization of cognitive changes in post-COVID patients with depression, we recruited a group of post-COVID patients newly diagnosed with depression and applied standard psychometric tests to that group of patients, as well as to matched controls. The use of standard tests will also help to define the general and specific features of PCD more clearly. The relationship between post-COVID depression, cognitive impairment, and the impacts of gender and age need to be clarified. 

The present study aimed to evaluate cognitive function in patients with clinically diagnosed post-COVID depression (PCD) using objective neuropsychological testing and associations between COVID-19 parameters and cognitive impairment in PCD. The current study brings new insights to understanding clinically diagnosed post-COVID depression and related cognitive impairment, and helps to find contributing factors to the severity of the disease consequences.

## 2. Materials and Methods

### 2.1. Study Participants and Clinical Assessment

The study participants (n = 109) were recruited by the Mental Health Research Institute (Tomsk, Russia), Medica Diagnostic and Treatment Center (Tomsk, Russia), and Tomsk State University (Tomsk, Russia) between September 2022 and June 2023. The inclusion criteria were the following: an age from 18 to 61 years, the absence of a history of traumatic brain injury, and the absence of any diagnosed neurologic or psychiatric condition prior to COVID-19. The exclusion criteria were previous positivity for COVID-19 (except for the control group) and self-withdrawal from the study. Written informed consent was obtained from all participants. The study design was approved by the local Ethical Committee of the Mental Health Research Institute (Tomsk, Russia, protocol No. 15/8.2022) and Bioethics Committee of Tomsk State University No. 12/06.2022, Tomsk, Russia) following the guidelines of the Declaration of Helsinki.

The Hospital Anxiety and Depression Scale (HADS) [19] was used to screen for symptoms of anxiety and depression. All subjects were assessed by a clinical psychologist, and those who scored higher (>8) on the HADS were assessed by a psychiatrist. A group of patients with affective disorder was formed by a psychiatrist based on a structured clinical interview for ICD-10 and a baseline assessment report, including socio-demographic characteristics, medical history, a questionnaire regarding COVID-19, and clinical and psychometric examination. The severity of the current depressive episode was assessed before the start of drug therapy using the Hamilton Rating Scale for Depression (HDRS) [20,21]. The total score is interpreted as follows: no depression (0–7); mild depression (8–16); moderate depression (17–23); and severe depression (≥24).

The individuals (n = 25) with diagnosed clinical depression (moderate depressive episode—F32.1, severe depressive episode without psychotic symptoms—F32.2, recurrent depressive disorder (first diagnosed at the time of the study), current episode moderate—F33.1, according to ICD-10) were included in the post-COVID depression (PCD) group. Participants (n = 46) with neurological complications of COVID-19 and without clinical depression were included in the comparison group (noPCD group). The first control group (n = 19) included healthy volunteers who were not COVID-19-positive and had not experienced symptoms of COVID-19 from the start of the pandemic until the time of examination. The second control group (ControlPC, n = 18) was formed from volunteers who had suffered from COVID-19 but did not experience post-COVID symptoms at the time of the research. The demographic characteristics of the participants are shown in Table 1. The groups did not differ significantly in age, gender, education, and severity of COVID-19 (PCD and noPCD groups) according to Chi-square criteria.

### 2.2. Questionnaire to Assess Acute and Post-COVID Symptoms

All participants, except for the control group, filled out a COVID-19 questionnaire. The questionnaire included questions about the number, severity, and date of illnesses, the PCR tests, vaccination, and symptoms of the acute and post-COVID phases. As symptoms of the acute phase, patients were asked to note the presence or absence of anosmia, ageusia, fever, difficulty breathing, cough, muscle weakness, myalgia, headache, and dizziness. As symptoms of the post-COVID phase, patients were asked to note the presence or absence of headache, dizziness, brain fog, anosmia, ageusia, sensitivity, hypertensia/hypotensia, insomnia, fatigue, attention and memory deficit, myalgia, depression, and panic attacks. Based on the results of the answers, the numbers of symptoms in the acute and post-COVID phases were calculated as the sum of symptoms (1 symptom—1 point), for which positive answers were given for all diseases. Number of symptoms has proven itself well as an independent predictor of post-COVID complications and for assessing the severity of post-COVID [22,23,24,25].

### 2.3. Neuropsychological Assessment

All participants were evaluated with several psychometric tests. The procedure was carried out by a clinical psychologist. The psychometric testing included the Montreal Cognitive Assessment (MoCA) [26], the Word Memory Test (WMT) [27,28], Trail Making Test, (TMT) [29], Stroop Color Word Test (SCWT) [30,31], and olfactory test. 

#### 2.3.1. Montreal Cognitive Assessment (MoCA)

The Russian version [32] of the Montreal Cognitive Assessment (MoCA) test [26], version 7.1 [33], is used for global assessments of cognitive function. Within the MoCA test (30 points maximum), 7 indexes [33] evaluate visuospatial/executive abilities (0–5 points), naming (0–3 points), attention (0–6 points including forward and backward digit span (0–2), vigilance (0–1), and calculation (0–3 points)), language (0–3 points), abstraction (0–2 points), short-term memory (0–5 points), and orientation of time and place (6 points). A total score of 25 and more is classified as normal, while 25 or less as cognitive impairment [26,32]. The MoCA and its subtests were validated on MDD patients and showed a high reliability [34].

#### 2.3.2. Olfactory Testing

Olfactory testing was performed with Sniffin’ Sticks Test kit (Burghart Messtechnik GmbH, Holm, Germany) [35,36]. Screening Sniffin’ Sticks Test is the most standardized test for smell disorder detection and showed a high sensitivity (93.4%) and specificity (68.2%) in COVID-19 patients [37]. The subjects sequentially identify 12 smells from a standardized set of well-known odors (coffee, orange, garlic, and cloves, etc.), making a choice from 4 proposed options. The identification version of the test was used. The odor was presented for 3 s and the pause between the presentation of odors was 30 s. The number of correct answers was counted.

#### 2.3.3. Stroop Color Word Test

Cognitive control was measured using the Russian version of the classical Stroop task [30,31], as modified by Cousijn et al. [38]. It is one of the most widely used tests that has been validated and shown to be reliable [39]. The test consisted of three subtests. The material for each subtest was one sheet of white paper, on which 100 words or single-color hexes were printed in random order. In the first subtest (word condition, W), the words were printed in black ink and meant four colors: “синий” (blue), “зеленый” (green), “красный” (red), and “желтый” (yellow). The participants had to read the words out loud as quickly as possible. In the second subtest (color condition, C), the participants saw solid-color hexes (blue, green, red, or yellow) and were asked to name the color. In the third subtest (word-color condition, WC), the printed words were related to the same four colors but were printed in a mismatched color (e.g., the word “blue” printed in red ink) and in matched colors. The total time in seconds spent completing each of the three subtests was measured. Additionally, an interference effect was determined by calculating the ratio between the times required for the W and C conditions (low interference) and the ratio between the times required for the CW and C conditions (high interference) [40].

#### 2.3.4. Word Memory Test

In the Word Memory Test (WMT) [27,28], 10 printed Russian unrelated words were presented to the participants. The Russian-language adaptation was developed and validated previously [41]. The participant was instructed to read and remember each word. After the presentation of the words, immediate recall was assessed. If the participant did not reproduce all the words, the psychologist tried to help them by providing associations with the missing word (assistance). About 15 min later, the participant was asked to reproduce the previously memorized words. After this, the psychologist tried to enable recall of the missing words using associations. Scores were assigned for immediately reproduced words (0–10 points), delayed words reproduced (0–10 points), and words additionally reproduced with the assistance of the psychologist. 

#### 2.3.5. Trail Making Test

The Trail Making Test (TMT), part A [29], was used to assess the speed of information processing. The participant was asked to connect 25 numbered circles in sequence (part A). The time spent on the task and the number of errors were recorded. Previously, this test was validated on MDD patients and showed a high reliability [42].

### 2.4. Statistical Analysis

Statistical analysis was performed using the Statistica 10.0 software. Differences between groups were analyzed using an analysis of variance (ANOVA) followed by post hoc Fisher LSD tests. The model included parameters of neuropsychological testing as dependent variables, group membership and gender as independent categorical factors, and age as a covariate. Differences in symptom frequencies between groups were analyzed using the Chi-square test. Associations between neuropsychological test results, depression, and COVID-19-related parameters were assessed using a multivariate multiple regression analysis. Only participants with a history of COVID-19 were included in the multiple regression analysis (PCD, noPCD, and ControlPC groups). 

To identify the independent factors affecting cognitive performance and to reduce the number of variables, the results of the cognitive tests (MoCA, WMT, SWCT, and TMT) were examined using a factor analysis. Seven independent factors with eigenvalues of >1 were identified. The individual factor scores were used as dependent variables for the multivariate multiple regression analyses.

The following independent variables were included in the single regression model as potential predictors of cognitive abilities:

(1) categorical predictors—sex (2 levels: male, female), diagnosed depression (2 levels: yes, no), and COVID-19 severity (2 levels: 1—mild, 2—moderate/severe/critical);
(2) continuous predictors included years of education, age, time after the first and last COVID-19 infection, and number of acute and post-COVID symptoms.

The Benjamini–Hochberg procedure for false discovery rate (FDR) correction was used to adjust the *p*-values to prevent false-positive results in all statistical tests involving multiple comparisons (intergroup differences in ANOVA and univariate results for each dependent variable in multivariate multiple regression analysis). Comparisons and models were considered statistically significant if the *p*-value was less than 0.05.

## 3. Results

### 3.1. Clinical Assessment of the Patients with Post-COVID Depression 

The patients who developed a depressive episode following a COVID-19 infection were combined into the post-COVID depression (PCD) group. Among 25 patients with PCD, 44% showed symptoms of atypical depression, such as an increased appetite, weight gain, sleeping more than 10 h, emotional reactivity, heaviness in the limbs, or chronic fatigue. Suicidal tendencies were identified in 52% of patients. The clinical characteristics of the patients with PCD are presented in Table 2.

### 3.2. Acute and Post-COVID Symptoms 

Group characteristics related to disease severity, time since first and last COVID-19 infection, and symptoms in the acute and post-COVID phases are presented in Table 3.

The symptoms in the PCD patients differed significantly from the ControlPC and noPCD groups in both the acute phase of the disease and the post-COVID phase. In the acute phase, the number of symptoms in the PCD group was significantly higher than that in the other studied groups. Ageusia and headache were checked in the questionnaire by patients in the PCD group more often in comparison with the ControlPC group. However, these differences between groups were borderline statistically significant. The patients from the noPCD group felt dizziness more often than the ControlPC group. The total number of acute symptoms in the noPCD group in the acute phase was also higher compared to the ControlPC group, while no differences between the PCD and noPCD groups were obtained. 

In the post-COVID phase, the differences in symptoms between the studied groups were more essential. In the PCD group, more than half of the patients experienced anosmia and ageusia (64% and 56%, correspondingly, *p* < 0.05 vs. the ControlPC group), while, in the noPCD group, only 29% and 21% reported these symptoms. Almost all patients in the PCD group reported sleep disturbances, fatigue, attention deficits, and depression. In contrast, half or fewer patients in the noPCD group reported these symptoms (*p* < 0.05 between groups for fatigue, attention deficit, and self-estimated depression). Both the PCD and noPCD groups differed significantly from the ControlPC group in insomnia, fatigue, and depression. The PCD patients also had symptoms of memory deficit, myalgia, and panic attacks more often than controls. The average number of symptoms of the patients in the PCD group was 1.3 times higher than that in the noPCD group and 2.8 times higher than that in the ControlPC group.

### 3.3. Results of Neuropsychological Testing 

Patients in the PCD group showed significantly higher scores on the HARS depression-related scales in comparison with both control groups and the noPCD group (Table 4). In addition, patients in the PCD group showed significantly higher insomnia (ISI total scores) when compared to controls, as well as to the noPCD group. Men and women in the PCD group showed similar differences to the comparison groups on these tests.

In the MoCA cognitive test, most indexes for the patients in the PCD and noPCD groups were lower than those in both control groups. The difference was statistically significant in the assessment of the total score (*p* < 0.05), only for female participants. Statistically significant differences were found in attention and visuospatial abilities between women in the PCD and control groups. Women in the noPCD group showed differences in language abilities from the controls.

The patients’ ages had a significant impact on the MoCA memory index and total scores: 16% of patients with PCD and 15% of noPCD patients had a marked decline in cognitive function with age, with a total score of less than 25 points. 

The Word Memory test (WMT) showed greater differences for male patients in the PCD group compared to the other groups. Thus, men in the PCD group had a significantly worse performance in both the immediate and delayed word recall tests compared to both control groups and the noPCD group. Women in the PCD group showed a significantly worse performance only in the immediate word recall test and only when compared to the control groups, without differences from the noPCD group. It should be noted that the noPCD group showed very little difference compared to the control groups (Table 4). The age effect was significant for the delayed recall and total scores. The sex effect on delayed recall and total scores was close to significant (*p* = 0.07 and *p* = 0.05), correspondingly. 

The Stroop Color Word Test (SCWT) revealed significant intergroup differences, only for males. Men in the PCD group showed longer processing times in the simple congruent W condition and more comprehensive CW condition when compared with the noPCD and control groups. The age effect was significant only for the W condition. 

The Trail Making Test (TMT) showed longer processing times in test performance only for female patients. Women in the PCD group performed the TMT task slower in comparison to the noPCD patients and patients in both control groups. However, the number of errors in this test for female participants with depression was significantly less than in the control group. The age effect was significant for processing time in the TMT test.

No differences between groups were found in the olfactory testing.

### 3.4. Associations between COVID-19-Related Parameters, Depression, and Neuropsychological Testing

A factor analysis was applied to identify independent factors of post-COVID changes in cognitive abilities. As a result, seven factors were identified that explained 75% of the variability in cognitive performance in the individuals with a history of COVID-19 (Table 5). The identified factors could be clearly interpreted and did not correlate with each other, which confirmed their unique contribution to the data variance. As follows from Table 4, two factors were associated with the MoCA parameters, three factors were associated with the memory test, one factor was associated with the SWCT low and high interference indexes, and one factor was associated with the processing times in the TMT and SWCT tests.

Based on the results of the factor analysis, we developed a multivariate multiple regression model to predict cognitive performance in the MoCA, WMT, SWCT, and TMT tests (Table 6). Three of the seven factors had significant multiple regression equations within this multivariate model: factors 1, 2, and 6. The six identified significant predictors within the model were education, age, diagnosed depression, the number of post-COVID symptoms, the interaction of COVID-19 severity and diagnosed depression, and the interaction of sex and diagnosed depression.

Level of education was the only significant predictor (*p* < 0.001) of factor 1 associated with the MoCA total scores, attention, and language subtests.

For factor 2, associated with memory scores, the significant predictors were age, diagnosed depression, the number of post-COVID symptoms, and the interaction of sex and diagnosed depression. The highest significant predictors (*p* = 0.002) of memory impairment were diagnosed depression and the interaction of sex and diagnosed depression. The contributions of age and number of post-COVID symptoms to the prediction of memory impairment were also significant (*p* < 0.05). A worse performance was associated with diagnosed depression, more post-COVID symptoms, older age, and male sex.

For factor 3, related to processing times in the TMT test in the simple congruent task in the SCWT test, the main predictor was age (*p* = 0.004). Specifically, older participants took longer to complete the task. Two other significant predictors were diagnosed depression and the interaction of COVID-19 severity and diagnosed depression (*p* < 0.05). Depressed patients, especially those with a more severe course of the disease, showed longer processing times.

For the other factors of cognitive abilities, a significant regression equation predicting the results could not be constructed.

## 4. Discussion

The unique characteristic of the current study is the application of standard psychometric tests to post-COVID patients with diagnosed depression, as well as to matched control groups. This approach reduces subjectivity, increases precision in the assessment of cognitive changes, and helps to find contributing factors to the severity of the disease consequences.

### 4.1. COVID-19 as the Cause of Depression and Cognitive Impairments

In our study, objective psychometric tests revealed multiple neurocognitive changes in patients diagnosed with depression as a complication of COVID-19. Several changes were expected for the PCD patients, such as an increase in HDRS and HADS scores. A key finding was that the PCD patients showed significantly (adjusted to age) worse results in several cognitive tests, including declines in the MoCA, WMT, SCWT, and TMT. This impairment in cognitive abilities in the PCD group was more prominent not only in comparison with two control groups (Control and ControlPC), but also in comparison with a large group of patients with post-COVID-19 syndrome without diagnosed clinical depression (noPCD group).

The application of multivariate multiple regression helped to identify predictors that affected cognitive impairments in memory and information processing speed. Level of education was a single significant predictor of general intelligence (factor 1, which includes MoCA total scores).

**Figure 1:**
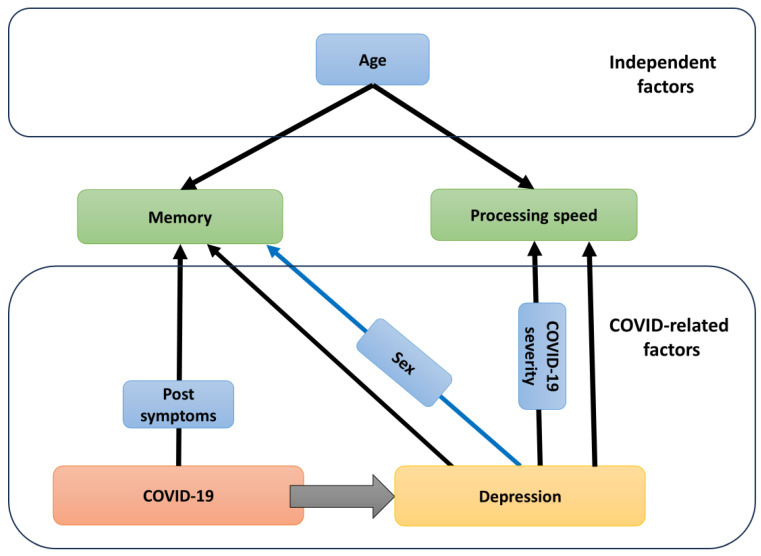
Scheme of the COVID-19-related and COVID-19-independent factors and their influence on cognitive abilities. Presence of depression increases cognitive decline in memory and information processing. Blue shapes indicate the predictors of cognitive outcomes. Black arrows indicate negative impact, blue arrow indicate the worst performance for men.

Figure 1 is a schematic representation of our study results that helps to summarize our findings. Diagnosed post-COVID depression itself significantly predicted memory and processing speed impairments. Post-COVID depression, in combination with male sex, predicted memory impairment. Post-COVID depression, in combination with a more severe course of COVID-19, predicted a lower processing speed. The number of post-COVID symptoms was also a significant predictor of memory impairment. Regardless of depression and COVID-19, older age predicted worse memory test results and processing speed.

The complications of COVID-19 may influence cognitive abilities directly and indirectly by causing depression. Direct influence is mediated by post-COVID symptoms. Decline in memory and information processing in post-COVID patients is more pronounced when COVID-19 causes depression. The severity of COVID-19 and male sex, combined with depression, are additional factors that impair memory and processing speed.

Despite multiple reports on cognitive and depressive impairments [2,3,4,5,6,7,8,9,10,11,12,43] persisting for a year or longer after COVID-19 recovery [8,44,45], no studies have shown cognitive impairment in patients with clinically diagnosed post-COVID depression.

Several studies have explored cognitive functions in patients with COVID-19-related depressive symptoms (not clinically diagnosed depression). Poletti et al. [6] investigated the cognitive function of COVID-19 survivors at 1, 3, and 6 months after recovery and compared the results to healthy controls and MDD patients without COVID-19. The study showed that 75% of COVID-19 patients had impairment in at least one cognitive function. However, psychomotor coordination and processing speed in COVID-19 patients were worse than those in healthy controls, but better than those in MDD patients. No difference between COVID-19 survivors and MDD patients was observed in verbal fluency and executive functions, but both groups showed lower results in those tests than healthy controls. No differences were found between COVID-19 patients and healthy controls in working memory and verbal memory. Pinnock et al. [46], in a prospective study of post-COVID patients, found a reduction in processing speed in favor of execution accuracy, deficits in complex attention and memory, and mild to moderate depression and anxiety symptoms at 1.5 years after recovery. Those findings were consistent with our results showing memory deficits in PCD patients and a decrease in processing speed in favor of accuracy in the TMT test.

Simonetti et al. [5] found an association between post-COVID-19 syndrome and mixed depression, i.e., a specific sub-form of depression characterized by a high level of excitatory symptoms. The group of depressed patients was formed only on the basis of the Hamilton scale. Unfortunately, this study did not examine cognitive impairments in PCD patients. Our results did not confirm the prevalence of excitatory symptoms in post-COVID patients, and oppositely, we found longer processing times in the TMT and SCWT compared to controls.

Our study brings novel insights on COVID-19-related consequences on mental health and identified predictors that affect cognitive impairment.

### 4.2. Comparison of Cognitive Changes in PCD Patients with Literature Data on MDD Patients

The COVID-19 pandemic significantly contributed to an increase in the rate of depressive episodes within MDD [47]. In our study, the criteria for defining the PCD group corresponded to the clinical criteria for diagnosing a depressive episode within MDD according to ICD-10. Therefore, it will be logical to compare our findings with studies of cognitive decline in MDD patients. Despite some similarities in symptoms, MDD and PCD have different causes: while MDD etiology is multifactorial [48], the main cause of clinical depression in PCD patients is COVID-19 infection.

Numerous studies have reported cognitive impairment in patients with MDD, including deficits in executive function, processing speed, memory, and attention (reviewed by [49,50,51,52]). A global assessment of cognitive abilities using the MoCA test also shows impairment in MDD patients [53,54,55,56]. About half of older patients with MDD scored below normal (25 scores or less) on the MoCA test [53,54], while a sample of patients with an age similar to our sample showed a lower percentage of cognitive decline [56]. Nyundo and Ismail [56] reported that 32.7% of MDD patients of similar age (mean age 42 years) had scores lower than 26 (the mean score was 26.56). Our results showed a similar mean score (26.48), but a smaller percentage (16%) of patients with scores less than 26. These differences might be likely explained by differences in the number of episodes and disease duration. In the study by Nyundo and Ismail [56], only 23% of patients had experienced 1–2 depression episodes and only 8% of patients had a disease duration of less than a year. In our study, the number of episodes in all patients did not exceed two and in 56% of patients, disease duration was less than a year. It should be noted that, in our study, the decrease in the total score on the MoCA test was mainly associated with decreases in the memory (3.56 in patients with DMD vs 4.05 points in controls) and attention (5.44 in patients with DMD vs. 5.84 points in controls) indexes. These results were confirmed by a significant decrease in the WMT test.

We observed sex-related differences in the total score on the MoCA test: women in the PCD and noPCD groups showed lower scores compared to controls. In contrast, Nyundo and Ismail [56] did not find gender differences in MoCA test performance in MDD patients.

The majority of published studies report memory deficits in MDD patients manifesting as immediate memory impairment. Xu et al. [57] found immediate visual memory impairment in patients in a depressed state and in remission compared to healthy controls. Shimizu et al. [58] reported both immediate and delayed verbal memory impairment in remitted MDD patients in comparison to healthy controls. Hammar et al. [57] also found that MDD patients showed a deficit in immediate word recall compared to healthy controls. Baune [59] found differences only in immediate but not in delayed memory. These findings are similar to our results, which showed a greater impairment in immediate (both men and women) than delayed (men only) verbal memory recall in PCD patients compared to the control noPCD group. In contrast, Jia [60] showed that first-episode drug-naïve depressive patients had deficits in delayed but not immediate memory. Hammar et al., in their review article [49], suggested immediate memory impairment as an impaired informational encoding, but not as a long-term memory deficit. Our results support this hypothesis. Based on our data, the PCD patients showed similar results in immediate (9.04 ± 1.40) and delayed (9.04 ± 1.65) word recall, while the healthy controls results were 9.21 ± 1.18 and 9.83 ± 0.48, correspondingly.

Our results showed that severity of COVID-19, along with gender and diagnosed depression, were significant predictors of immediate and delayed verbal memory recall. Therefore, memory, especially short-term memory, may be most vulnerable after moderate or severe COVID-19. The association of the severity of COVID-19 with long-term neurological consequences is well documented [16,22,25,61,62,63,64]. Miskowiak et al. [61] also showed the relationship between severity and cognitive changes in post-COVID patients. The authors hypothesized that cognitive sequelae of COVID-19 might be associated with the severity of lung damage and potentially restricted cerebral oxygen delivery.

Another distinctive feature of cognitive impairment in MDD is impairments in executive function and processing speed detected by the TMT and Stroop task [49,50,51,52,65]. We also found a significant increase in processing speed with a higher accuracy in the TMT in the PCD patients compared to controls. In the Stroop task, verbal fluency in the simple W condition and more complex CW condition was worse in the PCD patients compared to the controls and noPCD group. According to the literature, impairment in executive function in MDD patients is linked to the inhibition of automatic response in order to produce a less automatic task-relevant response [49,66]. This explains the higher processing speed and lower number of errors in MDD patients that we observed. Moreover, several studies have suggested that inhibition could be a trait marker in first-episode patients [49,66,67] that persists in long-term follow ups, as was shown by Schmid and Hammar in 10-year longitudinal studies [49,67].

The results of the current study on post-COVID patients were generally similar to the published data reporting cognitive changes in MDD patients in objective cognitive tests.

### 4.3. Gender Differences in Cognitive Performance

A distinctive feature of our results was the influence of gender on cognitive impairment in PCD. Cognitive decline manifested differently in men and women with PCD. Compared to the noPCD and control groups, men with PCD showed worse performances on the Stroop task, MoCA attention component, and memory test (in both immediate and delayed recall), while women with PCD showed a decline in MoCA total score, an increased processing time with less errors in the TMT, and worse immediate word recall. In addition, male sex, in combination with depression, was a significant predictor of memory impairment.

It is known that women are much more likely to be diagnosed with MDD. The genetic mechanisms of this phenomenon are being studied [68,69]. At the same time, in most studies [55,70,71,72,73], with the exception of a few [74], the influence of gender on cognitive decline was not revealed. In contrast, our study revealed a significant influence of gender on cognitive impairment in PCD patients. Our results also suggest that, although PCD is less commonly diagnosed in men, it causes greater deficits in memory.

From the point of view of gender differences during COVID-19 and its complications, there is a fundamental contradiction unexplained to date. In particular, men are more likely than women to experience a more severe course of COVID-19 [75,76,77], but women are more likely to experience post-COVID complications [16,22,25,62,64,77,78]. Some studies have suggested that male sex is a predictor of more severe cognitive impairment in post-COVID-19 [79].

The nature of the greater prevalence of post-COVID complications in women is still unclear. The increased risk of post-COVID complications in women during menopause [80] and gender differences in autoimmune response [75,81] are mainly discussed. A more severe acute phase of COVID-19 in men is mainly associated with a worse immune-inflammatory response [76,82], a lack of protective effect of sex hormones [81], and angiotensin-converting enzyme 2 (ACE2) receptor [75].

It can be assumed that it is the acute phase of COVID-19 that causes depression and more pronounced cognitive impairment in men compared to women. The reasons for these gender differences remain to be determined.

### 4.4. Possible Physiological Basis of COVID-19-Related Cognitive Changes in PCD Patients

One possible mechanism of cognitive impairment in PCD might be the disruption of connections between brain regions caused by neuroinflammation and demyelination due to COVID-19 [44,83,84,85,86,87,88,89,90].

Most researchers believe that neuroinflammation leading to impaired connectivity might be the main cause of cognitive impairment after COVID-19. Several studies have demonstrated neuroinflammation [83,84] as well as disrupted connectivity and demyelination [44,85,86,88] in post-COVID patients. Since microglial and astroglial reactivation lead to impaired oligodendrocyte functioning and renewal [91,92,93], demyelination or decreased remyelination might also play important roles in cognitive changes in PCD patients. An MRI study performed on the same group of PCD patients supported the hypothesis of brain demyelination after COVID-19 [94]. In that study, we used quantitative macromolecular proton fraction (MPF) mapping [95,96,97] that strongly correlated with myelin content [95,98,99,100,101]. The study showed more extensive brain demyelination in patients with post-COVID depression in comparison to controls and post-COVID patients without clinically diagnosed depression. Moreover, our study identified the demyelination of the inferior fronto-occipital fasciculus (IFOF) as the primary predictor of PCD presence and severity [94]. Anatomically, the IFOF connects early visual processing in the occipital lobe (cuneus and lingual gyri) and the parietal regions with frontal lobe regions [102,103], and also includes the connections between the cingulo-opercular and frontoparietal networks [103,104]. Therefore, the IFOF plays a critical role in semantic language processing, goal-oriented behavior, visual switching tasks, and executive function [102,103,104]. Based on these results, demyelination of the IFOF largely explains the results of the psychological tests that we found in the current study: impairment in visual verbal processing, interference in the Stroop task, an increased processing time in the serial connection test, and the immediate reproduction of words after reading them in patients with PCD.

According to the questionnaire, the PCD patients experienced a significantly greater number of post-COVID symptoms, as well as suffered more often from anosmia (hyposmia), ageusia (hypogeusia), insomnia, fatigue, attention and memory deficits, and panic attacks in comparison to the patients of the noPCD group, who also experienced post-COVID complications. These results are largely supported by objective test results documenting impairments in memory, executive function, and processing speed in post-COVID depression. The exception is the symptom of anosmia (hyposmia). In contrast to other studies [17,105,106], we did not reveal any differences between post-COVID patients and the controls in Sniffin’s Stick Test.

There are still too few studies to confidently state that memory deficits and lower information processing are specific cognitive impairments in patients with PCD. More research is needed to link post-COVID structural and functional brain changes with cognitive impairment and depression, as well as the sex-related features of these changes. Future directions, including an MRI study of demyelination and connectivity, functional MRIs, and EEG studies in combination with neuropsychological testing, could clarify the mechanisms underlying post-COVID-19 syndrome.

## 5. Conclusions

The present study is the first to examine cognitive impairment in patients with clinically diagnosed post-COVID depression using neuropsychological testing.

The PCD patients showed gender-dependent significant cognitive impairments in the MoCA, WMT, SCWT, and TMT in comparison with controls and post-COVID-19 patients without diagnosed clinical depression.

COVID-19-related direct (post-COVID symptoms) and depression-mediated (depression itself, male sex, and severity of COVID-19) predictors of decline in memory and information processing were identified.

The current study brings new insights for understanding clinically diagnosed post-COVID depression and COVID-19-related cognitive impairment. Many of these changes are gender specific. Our findings may help to personalize the treatment of depression, accounting for the patient’s sex and severity of previous COVID-19 disease.

## 6. Study Limitations

The study was conducted on a relatively small sample of studied subjects. The study did not include independent control samples of MDD patients without COVID-19 for detailed comparisons of cognitive impairments between groups. The sample of patients with PCD was not balanced by gender (more women than men), which may have influenced the results.

## Figures and Tables

**Table 1:** The demographic characteristics of participants of the study.

Parameter	PCD	NoPCD	ControlPC	Control
Sample size	25	46	18	19
Male (%)	4 (16)	17 (37)	7 (39)	8 (42)
Female (%)	21 (84)	29 (63)	11 (61)	11 (58)
Education, years ± SD	15.2 ± 1.9	15.9 ± 2.1	16.1 ± 2.4	16.4 ± 1.8
Age, years ± SD	37 ± 13.7	43 ± 10.4	43.7 ± 9.7	38.3 ± 10.3
Age, median (min–max)	42.0 (19–59)	43 (21–61)	42 (24–61)	39 (20–58)

**Table 2:** Clinical characteristics of the patients with PCD (n = 25).

Parameter	Mean ± SD
Hamilton score (HDRS)	18.36 ± 3.66
Age of manifestation, years	34.62 ± 13.96
Number of episodes	1.75 ± 1.75
Duration of last episode, months	8.27 ± 7.31

**Table 3:** The severity of COVID-19 and acute and post-COVID symptoms of participants of the study.

Parameter	PCD	noPCD	ControlPC	Statistics
Severity, mild/moderate/severe/critical (%)	88/8/4/0	63/17/15/4	66/28/0/1	
Number of COVID-19 episodes, mean ± SD	1.60 ± 0.71	1.65 ± 0.77	1.50 ± 0.51	F(2, 86) = 0.30, *p* = 0.74
Time after the first COVID-19, months ± SD	20.3 ± 8.2	21.8 ± 9.4	16.3 ± 6.4	F(2, 86) = 2.6, *p* = 0.08
Time after last COVID-19, months ± SD	13.1 ± 10.3	15.0 ± 10.5	9.8 ± 5.5	F(2, 86) = 1.8, *p* = 0.16
**Acute symptoms**				
Anosmia/hyposmia, n (%)	22(88%)	34(74%)	15(83%)	-
Ageusia/hypogeusia, n (%)	19(76%) *	27(59%)	8(44%)	-
Fever, n (%)	22(88%)	44(96%)	16(89%)	-
Difficulty breathing, n (%)	14(56%)	27(59%)	7(39%)	-
Cough, n (%)	22(88%)	32(70%)	13(72%)	-
Muscle weakness, n (%)	24(96%)	42(91%)	15(83%)	-
Myalgia, n (%)	20(80%)	30(65%)	10(56%)	-
Headache, n (%)	22(88%) *	34(74%)	11(61%)	-
Dizziness, n (%)	14(56%)	28(61%)*	6(33%)	-
Number of acute symptoms	7.24 ± 1.85 *	6.48 ± 2.21	5.61 ± 1.94	F(2, 86) = 3.28, *p* = 0.042
**Post-COVID symptoms**				
Headache, n (%)	7 (28%)	6(13%)	2(11%)	-
Dizziness, n (%)	10 (40%) *	22(48%) **	2(11%)	-
Brain fog, n (%)	14 (56%)	19(41%)	6(33%)	-
Anosmia/hyposmia, n (%)	16 (64%) *&	16(35%)	5(28%)	-
Ageusia/hypogeusia, n (%)	14 (56%) **&	12(26%)	3(17%)	-
Sensitivity, n (%)	3 (12%)	7(15%)	1(6%)	-
Hypertensia/hypotensia, n (%)	7 (28%)	23(50%) *	4(22%)	-
Insomnia, n (%)	20 (80%) ***	27(59%) ***	5(28%)	-
Fatigue, n (%)	24(96%) ***&	36(78%) **	8(44%)	-
Attention deficit, n (%)	23(92%) ***&&	29(63%) ***	4(22%)	-
Memory deficit,%	19(76%) ***	39(85%)	4(22%)	-
Myalgia, n (%)	15(60%) *	25(54%)	5(28%)	-
Depression ^1^, n (%)	24(96%) ***&&&	24(52%) **	2(11%)	-
Panic attacks, n (%)	5(20%) *	3(7%)	0(0%)	-
Number of post-COVID symptoms	8.04 ± 2.23 ***&	6.26 ± 2.95 ***	2.83 ± 3.24	F(2, 86) = 17.95, *p* = 0.000

Data are presented as mean ± SD. Significant differences relative the ControlPC group: *—*p* < 0.05, **—*p* < 0.01, and ***—*p* < 0.001. Significant differences between the PCD and noPCD groups: &—*p* < 0.05, &&—*p* < 0.01, and &&&—*p* < 0.001. ^1^ The “depression” parameter in the table refers to self-reported depression as indicated by participants on the questionnaire, as opposed to depression diagnosed by a psychiatrist.

**Table 4:** The results of neuropsychological testing.

Test	Parameter	Sex	PCD				noPCD			ControlPC	Control	Significance of Factors (Covariates), *p*-Value		
			Mean ± SD	*p*-Values, PCD vs.			Mean ± SD	*p*-Values, noPCD vs.		Mean ± SD	Mean ± SD			
				noPCD	ControlPC	Control		ControlPC	Control			Age	Sex	Group
**HADS**	Total score	m	22.75 ± 9.53	0.000 ***	0.000 ***	0.000 ***	10.94 ± 6.24	0.42	0.40	9.29 ± 3.77	6.13 ± 2.10	0.22	0.98	0.000 ***
		f	20.71 ± 7.17	0.000 ***	0.000 ***	0.000 ***	10.90 ± 4.92	0.50	0.05	7.82 ± 4.05	9.18 ± 3.77			
	Anxiety	m	13.00 ± 3.91	0.000 ***	0.000 ***	0.000 ***	5.71 ± 3.43	0.77	0.12	5.29 ± 4.23	3.50 ± 2.12	0.12	0.81	0.000 ***
		f	10.43 ± 3.04	0.000 ***	0.000 ***	0.000 ***	6.48 ± 4.04	0.09	0.24	4.45 ± 2.30	5.09 ± 2.47			
	Depression	m	9.75 ± 5.79	0.031 *	0.008 **	0.001 **	5.65 ± 2.98	0.22	0.04 *	3.71 ± 3.30	2.63 ± 1.60	0.81	0.82	0.000 ***
		f	10.47 ± 4.78	0.000 ***	0.000 ***	0.000 ***	4.51 ± 3.33	0.35	0.73	3.36 ± 2.77	4.09 ± 2.81			
**MoCA**	Total score	m	26.62 ± 1.70	0.56	0.33	0.19	26.41 ± 2.87	0.28	0.27	27.00 ± 2.45	27.63 ± 1.40	0.02 *	0.09	0.07
		f	26.75 ± 2.22	0.90	0.03 *	0.04 *	26.69 ± 1.85	0.03 *	0.04 *	28.27 ± 1.55	27.38 ± 1.77			
	Visuospatial/ executive abilities	m	4.75 ± 0.50	0.38	0.53	0.32	4.35 ± 0.93	0.84	0.77	4.43 ± 1.51	4.25 ± 0.71	0.99	0.50	0.95
		f	4.19 ± 0.93	0.13	0.08	0.04 *	4.55 ± 0.63	0.55	0.36	4.73 ± 0.47	4.82 ± 0.60			
	Naming	m	3.0 ± 0.0	1.0	1.0	1.0	3.0 ± 0.0	1.0	1.0	3.0 ± 0.0	3.0 ± 0.0	1.0	1.0	1.0
		f	3.0 ± 0.0	1.0	1.0	1.0	3.0 ± 0.0	1.0	1.0	3.0 ± 0.0	3.0 ± 0.0			
	Attention	m	5.25 ± 0.96	0.51	0.03 *	0.15	5.53 ± 1.01	0.20	0.11	5.86 ± 0.38	6.00 ± 0.0	0.21	0.78	0.04 *
		f	5.47 ± 0.75	0.36	0.34	0.09	5.28 ± 0.92	0.13	0.38	5.91 ± 0.30	5.73 ± 0.47			
	Language	m	2.25 ± 0.50	0.92	0.62	0.62	2.23 ± 0.92	0.42	0.55	2.00 ± 0.82	2.50 ± 0.76	0.73	0.90	0.77
		f	2.33 ± 0.66	0.15	0.32	0.61	2.00 ± 0.96	0.03 *	0.53	2.64 ± 0.50	2.18 ± 0.75			
	Abstraction	m	2.00 ± 0.00	1.00	1.00	0.34	2.00 ± 0.00	1.00	1.00	2.00 ± 0.00	2.00 ± 0.00	0.15	0.96	0.73
		f	1.95 ± 0.22	0.36	0.55	0.55	1.90 ± 0.31	0.17	0.17	2.00 ± 0.00	1.88 ± 0.35			
	Memory	m	2.75 ± 1.50	0.36	0.14	0.12	3.35 ± 1.50	0.35	0.31	3.86 ± 1.07	3.88 ± 1.25	0.002 **	0.03 *	0.18
		f	3.71 ± 1.31	0.40	0.39	0.29	4.00 ± 1.13	0.83	0.67	4.09 ± 1.22	4.18 ± 0.87			
	Orientation	m	5.75 ± 0.50	0.40	0.55	0.47	5.86 ± 0.33	0.84	0.95	5.86 ± 0.38	5.88 ± 0.35	0.94	0.15	0.86
		f	5.95 ± 0.22	0.87	0.68	0.68	5.97 ± 0.19	0.58	0.58	5.91 ± 0.30	5.91 ± 0.30			
**WMT**	Total score	m	15.75 ± 3.10	0.000 ***	0.000 ***	0.000 ***	18.83 ± 1.42	0.79	0.87	19.43 ± 1.13	19.13 ± 1.25	0.01 *	0.07	0.000 ***
		f	18.57 ± 2.46	0.57	0.14	0.38	19.24 ± 1.20	0.27	0.64	19.45 ± 0.82	19.09 ± 1.22			
	Immediate recall	m	6.75 ± 2.22	0.47	0.06	0.06	7.45 ± 1.33	0.50	0.53	8.43 ± 1.17	8.38 ± 1.06	0.23	0.47	0.04 *
		f	7.15 ± 1.50	0.11	0.57	0.01 *	8.00 ± 1.17	0.99	0.04 *	7.45 ± 1.81	8.45 ± 1.57			
	Immediate assistance	m	1.25 ± 0.96	0.32	0.68	0.75	1.94 ± 1.25	0.51	0.41	1.57 ± 0.79	1.50 ± 0.93	0.57	0.11	0.42
		f	2.10 ± 1.04	0.83	0.44	0.17	2.17 ± 1.31	0.53	0.11	2.45 ± 1.75	1.45 ± 1.37			
	Immediate total	m	8.00 ± 1.41	0.000 ***	0.000 ***	0.000 ***	9.59 ± 0.24	0.86	0.83	10.0 ± 0.0	9.88 ± 0.35	0.15	0.19	0.000 ***
		f	9.24 ± 1.14	0.04 *	0.01 *	0.01 *	9.94 ± 0.57	0.22	0.22	9.91 ± 0.30	9.91 ± 0.30			
	Delayed recall	m	5.25 ± 3.30	0.30	0.11	0.11	6.35 ± 1.97	0.35	0.34	7.14 ± 1.68	7.13 ± 2.42	0.02 *	0.05	0.29
		f	7.24 ± 1.61	0.57	0.96	0.66	6.93 ± 1.91	0.61	0.36	7.27 ± 2.00	7.55 ± 1.63			
	Delayed assistance	m	2.50 ± 1.91	0.65	0.82	0.69	2.88 ± 1.87	0.38	0.25	2.29 ± 1.11	2.13 ± 2.30	0.31	0.18	0.38
		f	2.05 ± 1.20	0.55	0.81	0.30	2.31 ± 1.34	0.81	0.11	2.18 ± 1.66	1.45 ± 1.13			
	Delayed total	m	7.75 ± 1.71	0.02 *	0.02 *	0.03 *	9.29 ± 1.05	0.80	0.93	9.43 ± 1.13	9.25 ± 1.16	0.01 *	0.10	0.07
		f	9.33 ± 1.53	0.78	0.62	0.72	9.24 ± 1.02	0.46	0.88	9.55 ± 0.69	9.18 ± 1.25			
**SCWT**	W, time (s)	m	65.00 ± 11.40	0.02 *	0.12	0.02 *	53.00 ± 8.28	0.44	0.77	56.14 ± 16.18	48.09 ± 6.20	0.000 ***	0.02 *	0.02 *
		f	53.57 ± 11.73	0.33	0.60	0.65	51.03 ± 6.30	0.18	0.36	55.36 ± 10.59	51.88 ± 9.79			
	C, time (s)	m	80.75 ± 23.68	0.12	0.37	0.36	66.47 ± 7.90	0.51	0.48	71.43 ± 16.72	71.50 ± 25.25	0.23	0.05	0.60
		f	62.52 ± 21.20	0.42	0.73	0.33	70.34 ± 15.17	0.31	0.10	64.36 ± 9.98	60.55 ± 14.74			
	CW, time (s)	m	156.75 ± 22.2	0.03 *	0.20	0.04 *	119.88 ± 22.34	0.34	0.98	132.57 ± 43.49	119.62 ± 52.08	0.26	0.002 **	0.21
		f	111.95 ± 33.1	0.23	0.64	0.33	122.17 ± 24.09	0.15	0.08	106.82 ± 16.33	103.55 ± 26.56			
	Low interference	m	0.82 ± 0.09	0.95	0.93	0.87	0.80 ± 0.11	0.96	0.88	0.79 ± 0.11	0.76 ± 0.15	0.27	0.50	0.86
		f	1.10 ± 1.40	0.06	0.33	0.25	0.75 ± 0.15	0.61	0.74	0.86 ± 0.11	0.82 ± 0.17			
	High interference	m	2.01 ± 0.36	0.79	0.85	0.68	1.80 ± 0.20	0.94	0.81	1.84 ± 0.27	1.65 ± 0.16	0.14	0.81	0.81
		f	2.33 ± 3.13	0.16	0.23	0.26	1.76 ± 0.27	0.89	0.95	1.69 ± 0.34	1.73 ± 0.31			
**TMT**	Processing time, s	m	36.50 ± 7.98	0.62	0.37	0.78	33.29 ± 8.20	0.52	0.81	30.00 ± 15.96	34.50 ± 11.46	0.000 ***	0.30	0.20
		f	42.52 ± 14.64	0.01 *	0.04 *	0.01 *	34.38 ± 11.46	0.49	0.85	37.18 ± 7.45	33.64 ± 5.90			
	Errors, mean ± SD	m	0.0 ± 0.0	0.15	0.25	0.17	0.47 ± 0.62	0.87	0.91	0.43 ± 0.79	0.50 ± 0.76	0.86	0.76	0.07
		f	0.14 ± 0.36	0.11	0.55	0.01 *	0.41 ± 0.63	0.50	0.13	0.27 ± 0.47	0.73 ± 0.65			
**SST**	Total score	m	9.50 ± 1.73	0.92	0.72	0.90	9.41 ± 1.23	0.53	0.75	9.86 ± 1.57	9.63 ± 1.30	0.28	0.66	0.85
		f	9.43 ± 1.03	0.81	0.72	0.79	9.32 ± 2.11	0.57	0.93	9.64 ± 1.57	9.27 ± 1.35			

Data are presented as mean ± SD. Significance level: *—*p* < 0.05, **—*p* < 0.01, and ***—*p* < 0.001.

**Table 5:** The factor structure of the results of cognitive tests in post-COVID patients.

Factor	Eigenvalue	% Total Variance	Cumulative %	Variables with Scores > 0.7
Factor 1	4.25	20.25	48.50	MoCA total score, MoCA attention, MoCA language
Factor 2	2.82	13.43	33.68	WMT total score, WMT immediate total, WMT delayed total
Factor 3	2.56	12.21	45.89	SCWT low interference, SCWT high interference
Factor 4	1.78	8.47	54.36	MoCA memory, MoCA orientation
Factor 5	1.64	7.80	62.16	WMT immediate recall, WMT immediate assistance
Factor 6	1.42	6.76	68.93	TMT processing time, SCWT processing time in W condition
Factor 7	1.28	6.11	75.04	WMT delayed recall, WMT delayed assistance

**Table 6:** The parameters of multiple regressions predicting cognitive performance in post-COVID patients.

Parameter		Factor 1 (MoCA Total Score, Attention, Language)		Factor 2 (Immediate, Delayed, and Total Scores in WMT)		Factor 6 (Processing Time in TMT and SCWT, W Condition)	
Multiple R		0.51		0.50		0.45	
Multiple R^2^		0.26		0.25		0.20	
Adjusted R^2^		0.20		0.17		0.12	
F		3.40		3.15		2.52	
*p*		0.0002		0.0003		0.01	
**Significant predictors in the model**	**Multivariate Wilks’ lambda, *p***	**β coefficient**	***p***	**β coefficient**	***p***	**β coefficient**	***p***
Education	0.001	0.47	0.0000				
Age	0.005			−0.25	0.02	0.35	0.004
Diagnosed depression	0.003			0.48	0.002	0.31	0.01
Number of post-COVID symptoms	0.03			−0.24	0.03		
Severity × Diagnosed depression	0.03					−0.30	0.02
Diagnosed depression × Sex	0.02			−0.37	0.002		

Results are presented only for those psychometric parameters for which significant multiple regression was obtained.

## Data Availability

Data are unavailable due to privacy or ethical restrictions.
